# Sex-Specific Insights into Etiology, Diagnosis, Treatment, and Prognosis in Significant Tricuspid Regurgitation: A Narrative Review

**DOI:** 10.3390/biomedicines12102249

**Published:** 2024-10-03

**Authors:** Mariana Sousa Paiva, Rita Reis Santos, Sara Guerreiro, Regina Ribeiras

**Affiliations:** 1 Cardiology Department, Hospital de Santa Cruz, Unidade Local de Saúde de Lisboa Ocidental, Carnaxide, 2790-134 Lisbon, Portugal; 2 Cardiology Department, Hospital dos Lusíadas, 1500-458 Lisbon, Portugal; 3 Cardiology Department, Hospital da Luz, 1500-650 Lisbon, Portugal

**Keywords:** tricuspid valve, tricuspid valve insufficiency, secondary tricuspid regurgitation, women, sex, gender

## Abstract

In recent decades, a burgeoning interest in tricuspid regurgitation (TR) has been prompted by a heightened awareness of its prevalence and the advent of dedicated percutaneous treatment approaches. Despite considerable understanding of its intricate anatomy and response to varying pressure and loading conditions, the impact of biological sex remains a subject of investigation. While TR typically afflicts more women, significant differences in TR etiology and post-treatment outcomes have not been conclusively established. This narrative review seeks to consolidate the latest evidence concerning sex-related nuances in anatomy, pathophysiology, diagnosis, treatment, and prognosis of significant tricuspid regurgitation. Through synthesizing this information, we aim to provide a comprehensive understanding of how sex may influence the management and prognosis of this condition.

## 1. Introduction

Recently, TR has gained recognition as a prevalent valvular heart disease [1,2]. In the Framingham Heart Study, the presence of any severity of TR (ranging from trace to more than moderate) was 82% in men and 85.7% in women [1]. Although initially considered to have a benign course, current evidence suggests that it is associated with increased morbidity and mortality [3]. With increasing age, the prevalence of significant TR increases, and in men and women aged > 70 years the prevalence of moderate and severe TR reaches up to 1.5% and 5.6%, respectively [1]. It is important to acknowledge that TR can be primary, resulting from intrinsic valve abnormalities, or secondary, due to right ventricular or tricuspid annular dilation [4,5].

One of the major drawbacks in TR used to be the fact that a significant proportion of patients were deemed unsuitable for surgery owing to their advanced age and comorbidities [6]. This panorama changed with the development of transcatheter tricuspid valve intervention (TTVI), a safe, effective, and minimally invasive therapeutic alternative [2].

Multiple studies have reported sex-related differences in the presentation and outcomes of patients with cardiovascular disease, including valvular disease and heart failure [7,8,9]. Specifically, women have been found to be older at presentation for intervention, have less clinical benefit after mitral transcatheter edge-to-edge repair (TEER), and have significantly higher mortality after aortic valve intervention for low-flow low-gradient aortic stenosis [7,10,11]. Conversely, studies regarding on dilated cardiomyopathy suggest that women who exhibit left ventricular reverse remodeling have better outcomes compared with their male counterparts [9].

Natural history studies have demonstrated an increased prevalence of significant tricuspid regurgitation (TR) in women [1]. Additionally, several risk scores, including the EUROscore II and the Society of Thoracic Surgeons’ tricuspid valve surgery (STS-TVS) score, which are validated to predict outcomes for isolated tricuspid valve (TV) surgery, include female sex as a risk factor [2,12,13,14,15]. However, the impact of sex on the characteristics and outcomes of patients with significant TR remains largely under-studied [16].

Hence, we sought to comprehensively analyze sex-related differences regarding the clinical presentation, anatomy, pathophysiology, diagnosis, treatment, and prognosis of significant tricuspid regurgitation. Of note, even though gender differences can impact prognosis, we focused our review on the biological term ‘sex’, usually categorized as male or female.

## 2. Materials and Methods

Electronic databases (MEDLINE and CENTRAL) were scanned from January 1990 to August 2024. We included the following listed keywords: “sex” or “gender” and “tricuspid regurgitation” or “tricuspid insufficiency” or “tricuspid valve disease” or “TR” and “transcatheter tricuspid valve repair” or “TTVR” or “tricuspid surgery” or “tricuspid valve replacement”. Only English manuscripts were considered.

## 3. Anatomy

The TV is located between the right atrium (RA) and the right ventricle (RV) and was originally described as having three leaflets (anterior, septal, and posterior) that insert into the annulus (TA) and are separated by three commissures (anteroseptal, posteroseptal, and anteroposterior) [17,18]. Furthermore, these leaflets are attached to one or more of the three papillary muscles (PMs) (anterior, septal, and posterior) through chordae tendinae [17]. The TV orifice, as viewed from the RA, has a semilunar shape and has a normal area of 7–9 cm^2^, corresponding to the largest cardiac valve [19].

Focusing on the leaflets, Rebecca Hahn et al. proposed a standardized nomenclature due to the variability of the number and location of supernumerary leaflets: type I: three-leaflet configuration; type II: two-leaflet configuration; type III: four-leaflet configurations; and type IV: five-leaflet configuration [20]. In her multinational retrospective study, only 54% of the patients had three well-defined leaflets, while 39% had four functional leaflets, with type IIIB (two posterior leaflets) being the most common among the latter. [20] No differences were identified based on sex [20].

Morphologically, the leaflets are thin, membranous, and vary greatly in size: the anterior leaflet is the largest in the radial direction, with the largest area and with the greatest motion; the posterior leaflet is the shortest circumferentially and may not be separated from the anterior; the septal leaflet is the shortest in the radial direction and is the least mobile due to a specific chordal type [17]. Another distinctive feature is the insertion of the septal leaflet, typically up to 10 mm more apically than the septal insertion of the anterior mitral valve (MV) leaflet. This specific anatomic feature allows the identification of the right ventricle, which is fundamental in congenital pathology [21].

Similarly to the MV, the TV subvalvular apparatus is composed of PMs and chords, albeit with a greater variability in number, length, shape, and arrangement [22]. The rough zone of the leaflets serves as the insertion point for the tendinous chords, which originate from the apices of conical PMs [21].

Whereas in the MV, we can observe two PMs (anterolateral and posteromedial) that provide chords to both leaflets, in the TV, there are three distinct PMs: the anterior (aPM), inferior (or posterior) (iPM), and septal PM (sPM) [23]. The aPM is the largest, with chordae supporting the anterior and posterior leaflets [24]. The iPM, often bifid or trifid, lends chordal support to the posterior and septal leaflets [24]. Finally, the septal leaflet, in the most frequent anatomy, receives chordae directly from the septum since the sPM is highly variable and formed by multiple muscular/tendinous strands attached to the leaflet [25].

Finally, the TA has a complex three-dimensional structure that undergoes dynamic changes throughout the cardiac cycle, exhibiting variability in shape and size [23,24]. It can be divided into two parts: a C-shaped ‘mural’ segment corresponding to the insertion of the anterior and posterior leaflets on the free wall of the RV, and a ‘septal’ segment, which is smaller and straighter, corresponding to the insertion of the septal leaflet [26]. For instance, there is no distinct fibrous ring to support the leaflets [26]. Its diameter is typically 30–35 mm, depending on body size [23].

**Figure 1:**
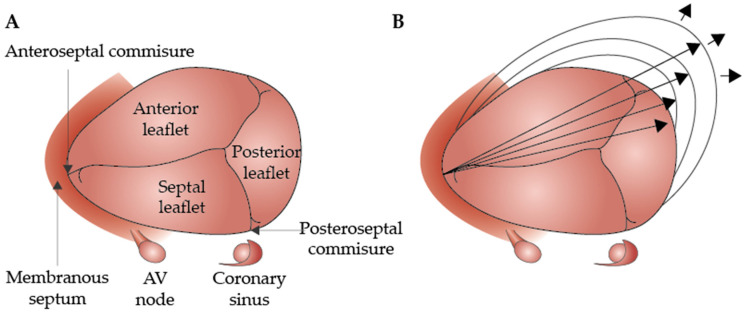
Anatomy of the normal tricuspid valve (**A**) and the outward dilation of the annulus toward the RV-free wall (direction indicated by the arrows) (**B**) (modified version of the original, reprinted with permission from Dreyfus et al. [27]).

During the cardiac cycle, the TA experiences an approximately 20% reduction in circumference during atrial systole [23]. In healthy individuals, it increases from mid-systole to early diastole, decreases during mid-diastole, and then increases again in late diastole, showing a biphasic pattern with peaks in early and late diastole (Figure 1) [24].

Regarding sex-related anatomical differences, El-Busaid et al., in a postmortem study, revealed that myocardium fibers were present in all male annuli but consistently absent in female annuli [28]. As a result, men’s atrioventricular annuli are more elastic, cellular, and smaller relative to the heart’s weight [28].

## 4. Etiology and Pathophysiology

Given the delicate anatomy of the TV, it becomes particularly prone to regurgitation [5]. Significant TR enters in a vicious cycle: TR induces RV and atrial dilation, leading to annular dilation, which worsens TR. Moreover, significant TR can induce ventricular interdependency, with septal flattening, reducing right-sided stroke volume and consequently LV preload [18].

**Figure 2:**
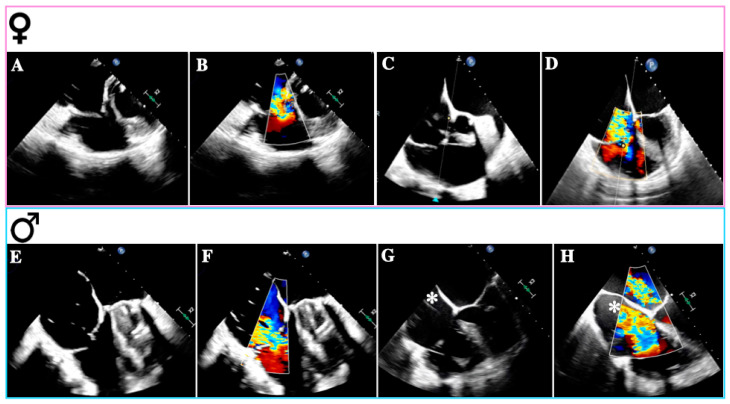
Causes of tricuspid regurgitation according to sex. Transesophageal echocardiogram—mid-esophageal views: (**A**): Rheumatic tricuspid regurgitation. (**B**): Rheumatic tricuspid regurgitation with color Doppler. (**C**): Atrial secondary tricuspid regurgitation. (**D**): Atrial secondary tricuspid regurgitation with color Doppler. (**E**): Ventricular secondary tricuspid regurgitation. (**F**): Ventricular secondary tricuspid regurgitation with color Doppler. (**G**): Lead-related tricuspid regurgitation. (**H**): Lead-related tricuspid regurgitation with color Doppler. The images (**A**–**D**) were obtained from female patients; (**E**–**H**) were obtained from male patients; * denotes the device-lead.

Primary TR (PTR) occurs in approximately 8–10% of all significant TR cases and has diverse etiologies, including congenital abnormalities, thoracic trauma, radiation therapy, drugs, infective endocarditis (especially in intravenous drug addicts), rheumatic heart disease, carcinoid syndrome, myxomatous disease, and endomyocardial fibrosis [22]. The mechanisms leading to TR vary depending on the underlying disease and typically involve structural damage such as leaflet perforation or restriction, commissural fusion, and chordal tethering [23]. While congenital TR, right-sided endocarditis, and carcinoid heart disease may be more prevalent in men, rheumatic heart disease is more common in women (Figure 2A,B) [29,30,31,32].

Secondary TR (STR) constitutes the majority of cases in our daily practice [19]. In these instances, valvular anatomy remains normal, but incomplete leaflet coaptation occurs due to leaflet tethering, tenting, and annular dilation, isolated or in combination [23]. It commonly arises from conditions inducing RV and/or RA dilation, such as left-sided heart disease, chronic atrial fibrillation (AF), and pulmonary hypertension (PH) [33]. Subtypes of STR include atrial STR (A-STR) and ventricular STR (V-STR) [33].

When assessing severe A-STR, it is important to evaluate the phenotypical phase of the disease because it has therapeutic and prognostic significance [34]. These phases are as follows: phase I, characterized by mild TA dilation; phase II, with moderate TA dilation/tethering; and phase III, with severe TA dilation/tethering [35]. They reflect disease progression, starting as A-STR and ending as V-STR [35]. In phases I and II, annuloplasty, coaptation, or both should be prioritized [35]. In phase III, it is crucial to evaluate RV function. If the RV is mildly to moderately impaired, valve replacement is recommended [35], However, if the RV is severely impaired with concomitant congestive heart failure, medical treatment is often the only option [35].

Firstly, A-STR is characterized by predominant TA dilation, resulting in leaflet-to-annulus imbalance as TV leaflets fail to cover the enlarged annulus area (Figure 2C,D) [35]. TA enlargement is asymmetric along the RV free wall, particularly in AF, where it occurs mainly towards its posterior aspect [36]. The primary trigger for TA dilation appears to be RA dilation and dysfunction associated with atrial arrhythmias (AF or atrial flutter) [22].

Additionally, unfavorable TA dynamics significantly contribute to A-STR [37]. Indeed, comparing AF with sinus rhythm (SR), TA area shortening is notably blunted due to the loss of atrial contraction, and the timing of TA minimal area is variable and frequently asynchronous concerning RV systole, resulting in a larger TA area during systole in most cardiac cycles [38]. Restoration of SR can effectively reduce A-STR [39].

A-STR is more prevalent in female patients, possibly due to a combination of AF-induced right atrial remodeling and sex-related differences in the structure of atrioventricular annuli as aforementioned [33]. Additionally, female sex hormones are believed to influence the development of more advanced atrial dysfunction and fibrosis, which are linked to atrial secondary mitral regurgitation and may also play a role in A-STR [9].

However, A-STR may manifest without AF, often in the presence of atrial myopathy and factors related to heart failure with preserved ejection fraction (HFpEF), such as aging, female sex, and left ventricular diastolic dysfunction [40]. RA myopathy can indeed precipitate isolated TR in a considerable proportion of patients without prior AF history (up to 38% in the European Society of Cardiology—Heart Failure Association, Heart Failure Long-Term Registry) [41,42], ultimately leading to ventricular remodeling with tricuspid leaflet tethering and further right ventricular dysfunction [43].

In the scenario of V-STR, RV remodeling predominates, resulting in dilation, elongation, and increased sphericity, which leads to leaflet tenting (Figure 2E,F) [4]. Gual Capllonch et al. found that increased tenting height was an independent predictor of significant V-STR in men but not in women [33]. Additionally, tricuspid regurgitant volume was higher in males than in females, possibly indicating greater disease severity in males [30]. Nonetheless, these volume differences may be attributed to variations in body surface area (BSA) between sexes [44]. Moreover, males exhibited lower baseline LVEF and TAPSE compared to females [33]. These findings suggest that despite a lower TR prevalence in males, they may experience more severe TR than females [45].

Once again, regarding HFpEF, which is more frequent in elderly women, there is growing evidence that it can lead to V-STR as a consequence of elevated left-sided pressures and post-capillary pulmonary hypertension [43].

It is noteworthy that PH is commonly linked with STR [19]. Gual Capllonch et al. showed that elevated systolic pulmonary artery pressure (SPAP) in females may contribute to the increased incidence of severe STR [33]. Still, Multak et al. found that most individuals with elevated SPAP had only mild TR, suggesting that other factors may also play a crucial role in determining STR severity [46].

Other significant triggers for female patients include left valvular disease and prior cardiac surgery, probably in relation with the increased incidence of rheumatic heart disease in this subgroup, as well as the delayed diagnosis of these patients, which allows time for left-sided disease to progress leading to TR and right-sided dysfunction [9,15,33,47]. Conversely, TR related to LV systolic dysfunction is more common in males [48].

A final consideration should be given to cardiac implantable electronic devices (CIEDs) and TR (Figure 2G,H) [49]. While CIED-associated TR is common, with a prevalence of 7–30% across several studies, a causal relationship should be suspected when TR emerges or worsens following right ventricular (RV) lead insertion (CIED-related TR) [50]. Previously classified as a “primary” cause of TR, the presence of a lead across the TV and the diverse mechanisms of TR in the context of a CIED have led researchers to reclassify CIED-related TR as a distinct etiological entity [50].

The mechanisms underlying CIED-related TR can be categorized as CIED-related TR (10–15%); LTR-A (causative) and LTR-B (incidental) [51]. When causative (LTR-A), TR results from the direct damage induced by the lead either by impinging, adhering, or perforating/lacerating a leaflet or interfering with the subvalvular apparatus [51].

The natural course of CIED-related TR is similar to other TR phenotypes, resulting in right heart remodeling characterized by increased RA and RV volumes, along with impaired RV function [52]. To date, no studies have reported any differences in CIED-related incidence between sexes. Imaging is crucial for determining the appropriate treatment approach, which should be decided by a dedicated multidisciplinary team [53].

## 5. Diagnosis

Echocardiography is the main imaging modality for assessing TV disease, providing information on anatomy, etiology, mechanism, and severity [54]. It also helps to detect its impact on RV size and function and to guide therapeutic strategy selection [54,55].

Compared to cardiac magnetic resonance imaging (CMR) and computed tomography (CT), transthoracic echocardiography (TTE) has superior temporal resolution, allowing it to better assess leaflet function and detect extrinsic interferences caused by masses and leads [54]. CT should be used to evaluate valve anatomy and the RV when considering percutaneous prosthesis and ring implantation therapeutic strategies [55].

CMR can help to quantify TR volume, but it is particularly useful to characterize RV volumes and function [54,56].

Transesophageal echocardiography (TOE) may be necessary if TTE imaging quality is insufficient. However, it is mandatory before percutaneous interventions, including transcatheter edge-to-edge repair (TEER), prosthesis implantation, or annuloplasty to better define the therapeutic strategies through a better-detailed valve anatomy and the assurance of enough image quality to guide the intervention [55].

Three-dimensional echocardiographic (3D) imaging accurately measures valvular lesions before surgery or transcatheter approaches, identifying morphologic predictors to guide treatment and reduce morbidity and mortality [55,57].

On two-dimensional (2D) TTE, the tricuspid valve is routinely recorded from the long and short-axis parasternal, apical four-chamber, and subcostal views [53]. The 2021 ESC guidelines for the management of valvular heart disease grade the severity of TR based on an integrative approach considering multiple qualitative and quantitative parameters by means of TTE, such as Doppler vena contracta (VC) width, proximal isovelocity surface area (PISA) radius, and effective regurgitant orifice area (EROA) [55].

Severe TR is identified by Doppler hemodynamic parameters, which include dense/triangular Doppler continuous wave jet contour with early peaking, VC width > 7 mm, PISA radius > 9 mm, EROA ≥ 40 mm^2^, and regurgitant volume ≥ 45 mL/beat [53]. Right chamber dilation is an important corroborative sign [55]. Hepatic vein systolic flow reversal, although rather insensitive, is a quite specific sign for severe TR [55].

A new classification for TR was proposed in a consensus document of the “Tricuspid Valve Academic Research Consortium Definitions for Tricuspid Regurgitation and Trial Endpoints”, subdividing the severe category into three subcategories: severe (VC width (biplane average) 7–13 mm, EROA by PISA 40–59 mm^2^, and 3D VC area or quantitative Doppler EROA 75–94 mm^2^); massive (VC 14–20 mm, EROA 60–79 mm^2^, and 3D VC area or quantitative Doppler EROA 95–114 mm^2^); and torrential (VC ≥ 21 mm, EROA ≥ 80 mm^2^, and 3D VC area or quantitative Doppler EROA ≥ 115 mm^2^) [51]. This proposal was based on the evidence of the data from trials and registries of TR percutaneous intervention, in which a two-grade reduction in TR, such as from torrential to severe, can still result in significant clinical benefit [51].

An important point not yet addressed in valvular guidelines is the fact that female patients generally have smaller cardiac and valvular dimensions than male patients. Recent research has emphasized the importance of using sex-specific measurements when grading valvular disease [7,58].

Because current valvular disease severity assessments are based on chamber volume cut-offs primarily derived from male populations, female patients are often underdiagnosed and under-referred for intervention due to their inherently smaller chamber and annular dimensions [7,59].

Nevertheless, clinicians should maintain a critical perspective regarding tricuspid evaluation in order to prevent potential overdiagnosis, especially for male patients, which, while not a significant concern at present, may emerge as an issue in the future.

## 6. Treatment and Prognosis

Treatment of tricuspid valve disease depends on the severity and underlying mechanism and has advanced rapidly in recent years. Despite this, there are no available sex-specific data regarding TR treatment.

Diuretic therapy is considered the cornerstone for symptomatic relief and for upstream organ protection, specifically for the liver, kidneys, and intestines; however, the European guidelines do not consider it as a Class I indication in any scenario [55].

Conversely, current guidelines recommend that patients with severe tricuspid regurgitation undergoing left-sided valve surgery or symptomatic patients with isolated severe primary tricuspid regurgitation without severe RV dysfunction should be referred for surgery. Guidelines also recommend that surgery should be considered in patients with mild or moderate secondary tricuspid regurgitation with a dilated annulus (≥40 mm or >21 mm/m^2^ by 2D TTE) undergoing left-sided valve surgery [55].

Traditionally, isolated tricuspid surgery has been associated with high operative mortality over time (9–10%) and it is presumed that most of these high-risk patients are referred for surgery too late, with RV dysfunction and end-organ damage, in opposition to left-sided valve disease [60]. Further, there are no randomized trials comparing surgery with medical therapy for severe TR. Nevertheless, recent data suggest that outcomes from surgery on TR may be better than previously observed, despite a relatively small number of cases and registry data. TV repair also appears to be associated with a lower in-hospital mortality than TV replacement (4.7% vs. 12.6%) [61,62].

The majority of studies that have evaluated isolated TV surgery have shown an equal or slightly higher number of female patients (approximately 50–55% of women) [61]. However, there was a predominance of men in the main randomized trial of patients undergoing concomitant TV intervention in mitral valve surgery, with 75% of male patients included in total [63]. The etiology of MR in the aforementioned trials was degenerative, which could explain the gender difference, since degenerative etiology is known to be more prevalent in men [64]. Furthermore, there was no statistically significant difference between men and women in both in-hospital and later mortality among patients who underwent surgical treatment, based on data from a recent meta-analysis [45]. The main difference in outcomes in this meta-analysis was a significantly higher need for permanent pacemaker implantation in male patients (RR: 1.45, 95% CI (1.10, 1.93), *p* = 0.010) [45].

TTVI is emerging as a promising new treatment and appears effective for patients deemed unsuitable for surgery [65]. It is already mentioned as a class IIB indication for the treatment of symptomatic inoperable severe STR in the latest guidelines [55]. Currently, there are several available techniques, ranging from TEER approaches to the percutaneous biological prosthesis, EVOQUE™, which has received CE mark approval in 2023 and United States Food and Drug Administration approval in 2024 [65,66,67]. Additionally, the bicaval prosthesis TricValve™ represents the final step before palliative care [68]. It is noteworthy that the presence of severe PH (i.e., ≥70 mmHg) and precapillary PH preclude any of those interventions [69].

TEER stands out as the technique with the most comprehensive and extensive data to date and the only one that has been evaluated in a large randomized controlled trial. Gender distribution was similar between the two main TEER trials, with 54% female for the TriClip™ and 55% female for the PASCAL© device [69,70]. In the TRILUMINATE pivotal trial, patients were enrolled for the TriClip™ system with symptomatic severe TR and high or intermediate risk for surgery, a majority of them with secondary TR (90%). TEER was associated with TR severity reduction and improved quality of life compared with medical therapy [69]. Additionally, the magnitude of the change in KCCQ score appeared to favor TEER for both genders, although this benefit seemed to be even higher for male patients (OR 3.3 in men and OR 2.49 in women). The reason for these results is not clear and no prespecified analysis was performed [69].

While in the TRILUMINATE study (a prospective, single-arm trial which evaluated TEER in symptomatic patients with moderate or severe TR and high surgical risk) there were fewer hospitalizations for both genders, in the TRILUMINATE pivotal trial (a prospective randomized study—1:1 TEER vs. medical therapy), it was not possible to show a reduction in hospitalizations when comparing TEER with medical therapy alone [69,71].

Moreover, the other TTVI techniques, such as EVOQUE™, the Cardioband™ tricuspid system, and TricValve™, had also been evaluated for TR treatment; however, they had a higher proportion of female patients enrolled in comparison with TEER studies (71–81% of women)—Table 1 [70,72,73]. These data align with our knowledge of tricuspid disease, where its prevalence is lower in men, mainly in the older age group [74]. In addition, in the TRISCEND trial (a prospective, single-arm study which evaluated transcatheter TV replacement in symptomatic moderate or severe TR), more patients were presenting in NYHA III-IV (i.e., NYHA III/IV: 75.4% in TRISCEND vs. 59.4% in TRILUMINATE), with chronic renal disease (58.5% vs. 35.4%), and with prior-year HF hospitalization (40.9% vs. 25.1%) compared to TEER studies [70]. Thus, a higher female sex prevalence and more advanced disease were observed in the transcatheter prosthesis study, despite the average age being similar between TTVI trials. Lastly, a propensity score analysis showed that TTVI in high-risk patients (including TEER, annuloplasty, and TricValve™) with symptomatic severe TR substantially reduced the incidence of 1-year mortality or HF re-hospitalization in men, independently of other factors, compared with medical treatment alone [75].

Considering the above information, several questions arise, and it remains to be clarified whether women had more severe disease at the time of intervention due to later referral, and whether men benefit more from TV intervention because of earlier referral or other unrecognized factors. Despite specific data on this topic not being available, we speculate that the predominance of atrial FTR in women and its slow progression might explain a later referral for TV intervention and possibly worse outcomes for women. However, randomized clinical trials focused on this gender difference are needed to draw further conclusions and for a better understanding of this topic.

## 7. Future Directions

Despite the rapid increase in knowledge on TR in recent decades, several gaps persist, particularly regarding sex differences in pathophysiology, management, and outcomes [76]. It is important to acknowledge that our review primarily includes observational studies that are subject to inherent biases, such as selection and measurement errors. Consequently, the findings presented may not fully encapsulate the complexity of sex-related differences in TR.

**Figure 3:**
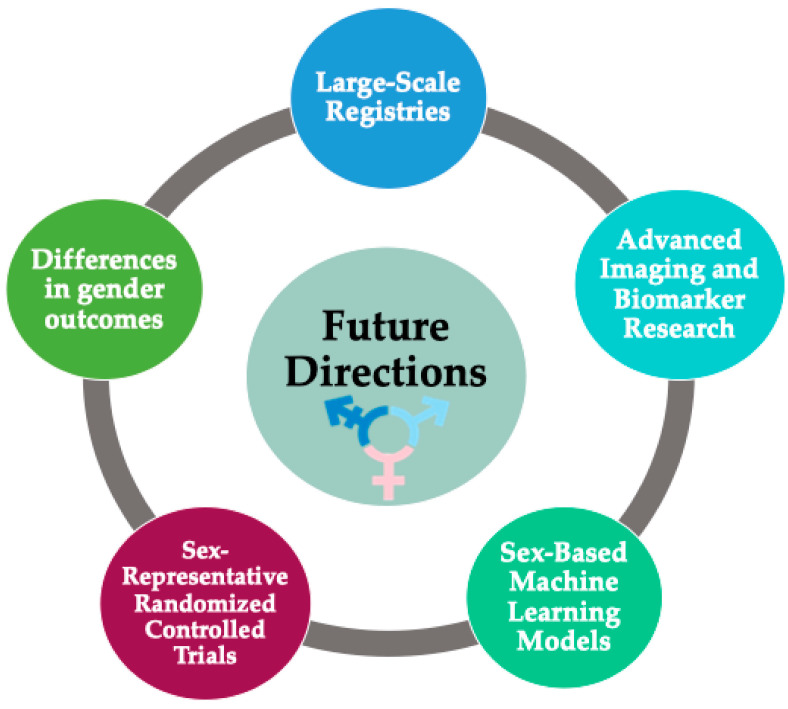
Future directions (suggested research priorities).

To address these limitations, we propose the establishment of large-scale registries to characterize the incidence and prognosis of TR more accurately across diverse genders and backgrounds (Figure 3). Such registries could facilitate the validation of diagnostic cut-offs designed to quantify the severity of regurgitation.

Future cardiac magnetic resonance imaging studies, biomarker research, and biopsy approaches may elucidate the differential roles of genetics, fibrosis, and related cellular processes in the tricuspid annulus, right atrium, and right ventricle between sexes [44,56].

Additionally, sex-based machine-learning models could be developed first to identify individuals at higher risk of progression, then to predict morbidity and mortality, and finally to determine the optimal timing for referral for further treatment [77].

Furthermore, there is a critical need for more randomized controlled trials that accurately represent both sexes, allowing for a deeper exploration of the efficacy of emerging treatments and the differences in outcomes among individuals receiving these interventions.

Lastly, there is a growing awareness within our community about the importance of respecting gender differences in a patient-centered approach, as highlighted in the 2024 European Society of Cardiology guidelines for the management of atrial fibrillation, which removed the sex category from the CHA2DS2-VA risk score [78]. Future research will provide more knowledge about the biological effects of synthetic hormones and improve management and prognosis across all gender identities.

## 8. Conclusions

Current knowledge on sex-related differences in TR is largely derived from observational studies, highlighting a critical gap in our understanding. Despite this, existing data underscore the importance of sex-specific considerations in tricuspid annulus anatomy, TR etiology phenotypes, diagnosis, and treatment.

Data suggest that AF and atrial myopathy contributing to A-STR affect women more, while V-STR is more common in men. Moreover, factors such as left valvular disease and prior cardiac surgery are significant triggers for TR in female patients, whereas TR related to left ventricular systolic dysfunction is more prevalent in males.

Women may present with more advanced disease at intervention due to non-diagnosed and insidious A-STR progression leading to late RV dysfunction. The fact that valvular guidelines do not account for smaller female cardiac dimensions is probably one of the multifactorial causes that prevent adequate diagnosis and early referral. The more advanced disease and later referral for women leads to under-intervention, less intervention benefit, and intervention denial.

Studies on TV surgery and TEER indicate gender distributions are similar, but men show greater benefits in TR severity and quality of life improvements. Again, the reasons behind these differences may include more advanced disease and later referral in women, but further investigation is necessary. It is crucial to conduct randomized trials to understand these sex differences better and to develop tailored management strategies for TR in both men and women.

## Figures and Tables

**Table 1:** Studies evaluating tricuspid regurgitation intervention.

	TricVALVE December 2019	TRI-REPAIR February 2021	Concomitant TV Repair in MV Surgery January 2022	CLASP-TR May 2023	TRILUMINATE May 2023	TRISCEND II December 2023	TRICUS January 2024
Trial characteristics	Propensity-matched registry (1:1) of patients who underwent TTVI with control cohort (medically managed patients with ≥moderate TR).	Single-arm, prospective study of Cardioband© tricuspid system in patients with ≥moderate and symptomatic TR despite medical therapy.	Prospective randomized trial of concomitant tricuspid annuloplasty in patients who underwent mitral-valve surgery for degenerative MR.	Single-arm, prospective of TEER in severe TR [PASCAL©].	Prospective randomized trial of percutaneous tricuspid TEER for severe TR [CLIP©].	Prospective, single-arm trial of transcatheter TV replacement (EVOQUE©) in patients with ≥moderate and symptomatic TR despite medical therapy.	Prospective, single-arm trial of the TricValve© system in TR patients, optimally medicated and ineligible for open heart surgery.
Gender	TTVI 55% women vs. Control 63% women	73% women	25% women	55% women	55% women	71% women	81% women
Baseline characteristics	268 pts (TTVI) 77 yo 93% NYHA class III or IV EuroSCORE II 12 ± 11%	30 pts 75 yo 83% NYHA class III or IV EuroSCORE II 4.1 ± 2.8%	401 pts 67 yo 30% NYHA class III or IV EuroSCORE II (no data)	65 pts 77 yo 71% NYHA class III or IV EuroSCORE II 5.0 ± 4.7%	350 pts 78 yo 57% NYHA class III or IV EuroSCORE II (no data)	176 pts 78.7 yo 75% NYHA class III or IV EuroSCORE II 5.1 ± 4.0%	44 pts 76 yo 100% NYHA class III or IV EuroSCORE II 5.8 ± 4.2%
TR etiology	90% functional	100% functional	No data	No data	94% functional	68% functional, 14% mixed	No data
Follow up	1 year	2 years	2 years	1 year	1 year	1 year	1 year
Main results	TTVI is associated with greater survival and reduced HF rehospitalization compared with medical therapy alone.	Cardioband system improved quality of life and exercise capacity; and resulted in sustained and significant TR reduction.	Tricuspid repair resulted in less frequent progression to severe TR and more permanent pacemaker implantation	Tricuspid TEER had sustained improvements in TR, functional status, and quality of life	Tricuspid TEER reduced the severity of TR, and was associated with an improvement in quality of life	Sustained TR reduction Increased stroke volume and cardiac output Improvement in clinical, functional, and quality-of-life outcomes	TricValve system associated with clinical improvements in terms of quality-of-life.

## Data Availability

The data underlying this article will be shared on reasonable request to the corresponding author.
